# Brief interventions for suicidal ideation in primary care: a systematic review

**DOI:** 10.1186/s12875-025-02848-4

**Published:** 2025-05-15

**Authors:** Puya Younesi, Carolin Haas, Tobias Dreischulte, Andrea Schmitt, Jochen Gensichen, Karoline Lukaschek, for the POKAL-Group, Puya Younesi, Carolin Haas, Tobias Dreischulte, Jochen Gensichen, Karoline Lukaschek, Markus Bühner, Peter Falkai, Peter Henningsen, Caroline Jung-Sievers, Helmut Krcmar, Gabriele Pitschel-Walz, Antonius Schneider, Katharina Biersack, Vita Brisnik, Julia Eder, Feyza Gökce, Lisa Hattenkofer, Lukas Kaupe, Jonas Raub, Philipp Reindl-Spanner, Hannah Schillock, Petra Schönweger, Victoria von Schrottenberg, Clara Teusen, Jochen Vukas, Kirsten Lochbühler

**Affiliations:** 1 Institute of General Practice and Family Medicine, University Hospital, LMU Munich, Nussbaumstrasse 5, Munich, 80336 Germany; 2 Graduate Program “POKAL - Predictors and Outcomes in Primary Care Depression Care” (DFG-GrK 2621), Munich, Germany; 3 Institute of Psychology, University of the Bundeswehr Munich, Neubiberg, Germany; 4 Department of Psychiatry and Psychotherapy, LMU University Hospital, LMU Munich, Nussbaumstrasse 7, Munich, 80336 Germany; 5 Laboratory of Neuroscience (LIM27), Institute of Psychiatry, University of Sao Paulo, São Paulo, SP 05453-010 Brazil; 6 Max Planck Institute of Psychiatry, Munich, 80804 Germany

**Keywords:** Suicidal behaviour, Suicidal ideation, Primary care, Brief interventions, General practitioner

## Abstract

**Background:**

General practitioners (GPs) play a crucial role in assessing and diagnosing suicidal ideation, often acting as the first person of contact for individuals with mental health concerns. Given the time constraints faced by primary care providers, interventions need to be brief and easily implemented. This systematic review seeks to identify, compare, and critically evaluate effective brief interventions for managing suicidality in primary care, offering a comprehensive overview and discussion of key findings.

**Methods:**

A systematic literature review was conducted using databases including MEDLINE, EMBASE, The Cochrane Library, PSYNDEX, and PsychINFO, supplemented by manual searches. Our search strategy focused on studies from 2000 to 2023. Risk of bias was assessed using the Cochrane RoB 2 Tool, and evidence quality was evaluated using GRADE, with adherence to the PRISMA-DTA checklist. A protocol was published in PROSPERO.

**Results:**

The search yielded 1248 publications. Of those, 44 were assessed for eligibility after screening, ultimately resulting in five included studies addressing four brief interventions for suicidality in primary care. Motivational interviews, safety planning, structured follow-ups, and collaborative care models were identified as key elements for future interventions to enhance the role of primary care in suicide prevention.

**Conclusion:**

This review highlights the need for further research to adapt brief interventions for primary care suicide prevention. Given their central role in patient care, GPs are well-positioned to identify and support individuals at risk. While initial promising approaches have emerged, further research in primary care suicide prevention is needed, and interventions tailored to the GP setting must be developed.

**Supplementary Information:**

The online version contains supplementary material available at 10.1186/s12875-025-02848-4.

## Introduction

Suicidality remains a critical global health and social issue, contributing to over 700,000 deaths annually and ranking among the leading causes of death worldwide [1]. Many countries have national suicide prevention strategies [2, 3] and some such as Ireland and Australia additionally have National Suicide Prevention Offices [4]. In Europe, EU-funded projects have advanced suicide prevention, such as European Alliance Against Depression (EAAD), Saving and Empowering Young Lives in Europe (SEYLE), Suicide Prevention Through Internet and Media Based Mental Health Promotion (SUPREME), and Reduction of Suicides and Trespasses on Railway Property (RESTRAIL) [5, 6].

Evidence-based prevention strategies identified in systematic reviews include restricting access to lethal means (e.g., firearms and barriers at high-risk locations), public education to reduce stigma, and targeted interventions for high-risk groups. However, it is highlighted in the literature that no single strategy can effectively address suicidality on its own: instead, a multi-level approach combining various interventions is essential for achieving significant reductions in suicide rates [7–9]. Pharmacological and psychological therapies play vital roles in these multi-level approaches, particularly when integrated into general practice [8, 10].

Mental health conditions, notably depression, anxiety, and schizophrenia, are closely associated with increased suicide risk [11]. Additionally, socioeconomic factors such as deprivation, unemployment, and social isolation significantly contribute to suicidality, with certain groups, including men, consistently exhibiting higher suicide rates than women [12–16]. Addressing suicidality effectively requires an understanding both individual and structural risk factors to guide comprehensive and impactful prevention strategies [17, 18].

The lifetime prevalence of suicidal ideation in the general population is estimated at 9.2% across countries [19].

Research also indicates that individuals in suicidal crisis are more likely to seek contact with general practitioners (GPs) rather than mental health services [20–23]. On the one hand, this indicates that individuals in primary care should be considered a key target population for suicide prevention efforts [18]. On the other hand, this presents a unique opportunity for GPs to provide support, sparking considerable research interest in their role in suicide prevention [24].

GPs hold a pivotal role in suicide prevention as they are often the first point of contact for individuals experiencing suicidal ideation [21]. GPs’ continuous, often long-term relationships with patients enable effective early detection, screening, and management of mental health issues, particularly when combined with psychosomatic care [7, 25–27].

Training and resources for GPs, such as screening tools for depression and suicidal behaviour, alongside appropriate interventions, have been shown to enhance their ability to manage suicidality effectively [8, 10]. However, high patient volumes and time constraints in primary care create challenges for delivering effective suicide prevention interventions [28–30].

Thus, there is a pressing need for interventions that are both effective and feasible within the time limitations of primary care. Ideally, such interventions should be brief, easy to implement, cost-effective, and require minimal staff resources [18], focusing on informing people about suicidal behaviour and motivating them to engage in safety planning and help-seeking, and problem solving.

However, there is no universally accepted definition of what constitutes a “brief” intervention, and various interpretations exist [31]. The authors of this review adopt the definition provided by National Institute for Health and Care Excellence (NICE), which states that a brief intervention includes discussion, negotiation, or encouragement, with or without written support or follow-up, and may also involve referral for further care [32]. Collaborative care between GPs and mental health and social professionals further supports suicide prevention efforts by ensuring holistic and continuous care, as well as developing practical strategies to manage future suicidal crises [1, 8, 10, 18].

This review critically assesses the effectiveness of brief interventions in primary care, with a specific focus on their capacity to enhance suicide prevention efforts within general practice. By evaluating the literature, the aim is to identify key components that could be integrated into primary care settings. This includes an exploration of practical interventions that align with the unique challenges of primary care, such as time constraints and high patient volumes. Ultimately, the review seeks to determine how brief interventions can be optimally designed and implemented to not only address the immediate needs of patients experiencing suicidal ideation but also to support sustainable suicide prevention efforts within general practice.

## Methods

The systematic review was conducted and reported following the Preferred Reporting Items for Systematic Reviews and Meta-Analyses (PRISMA) guidelines [33]. The study protocol was registered with the PROSPERO International Prospective Register of Systematic Reviews (CRD42023443026) [34].

### Search strategy

A comprehensive literature search was conducted across EMBASE, MEDLINE, PSYNDEX, PsychINFO und Cochrane Library for studies published from January 1, 2000, to October 31, 2023. Research from 2000–2023 better reflects current knowledge, ensuring modern methods and scientific standards. Recent studies increasingly utilise technologies like big data analytics, machine learning, AI, wearables, and sensors. Several German-language journals on suicidality target general practitioners. To include their crucial contributions, we chose German as a second language alongside English. Search terms focused on key concepts such as “primary care,” “suicide,” and “randomized controlled trial,” with keywords tailored to each database’s vocabulary. The complete search strategy is detailed in the supplemental material. The final search was conducted on 04 November 2023.

### Eligibility criteria

Only randomised controlled trials (RCTs) were considered. We excluded non-peer-reviewed publications, case studies, letters to the editor and studies of low methodological quality. The inclusion and exclusion criteria for the systematic review were carefully designed to focus on relevant studies that assess brief interventions for suicidality in primary care settings. Included studies involved patients aged 16 years or older who had either attempted suicide or experienced active or passive suicidal thoughts, provided that the intervention occurred in a primary care setting and were GP- or staff-led. Eligible interventions were defined as brief in nature and compared against care-as-usual, treatment-as-usual, or waitlist control groups. Excluded were studies involving patients younger than 16 years or with cognitive impairments or dementia.

### Study selection and data extraction

The search results were exported to EndNote [35], where duplicates were automatically removed. The remaining records were then transferred to “Rayyan”, a web and mobile app for systematic reviews [36], for a second deduplication process, conducted both automatically and manually by PY. The screening of titles and abstracts, along with the full-text review, was conducted independently and in a blinded fashion by two authors (PY, KL); eligibility was evaluated using titles and abstracts [36], and full-texts were subsequently screened based on predefined inclusion and exclusion criteria. Any disagreements at each stage were resolved through discussion among the authors (PY, KL, KB). Data extraction was independently conducted by two authors (PY,KL) using Microsoft Excel Sheet (Microsoft Corporation, 2018) based on the Cochrane Data Collection Form [37]. The extraction form captured details on study design, study sample characteristics (including sample size, country, and age), provider, setting, intervention lengths and frequency, intervention characteristics and main outcomes. If full-text articles were unavailable or data was missing, study authors were contacted directly.

### Outcome measures

#### Primary outcome

The primary outcomes were changes in suicidal behaviour-specific scores. Given the heterogeneity of primary outcomes and the limited number of studies, a qualitative data synthesis was preferred over a quantitative analysis. While a meta-analysis would have provided a basis for a quantitative summary of the data [38], a narrative synthesis was chosen instead to qualitatively interpret the findings. This methodological approach aims to systematically review the literature to comprehensively capture the current state of research on a specific topic [38].

### Quality assessment

The risk of bias assessment was conducted using the RoB-2 tool [39]. RoB-2 provides a methodological framework for the systematic evaluation of bias risks in the results of randomized studies. We also used the internationally recognized GRADE methodology [40, 41] for a systematic and transparent approach to assess evidence quality and to formulate clinical practice recommendations.

## Results

### Study selection

**Fig. 1:**
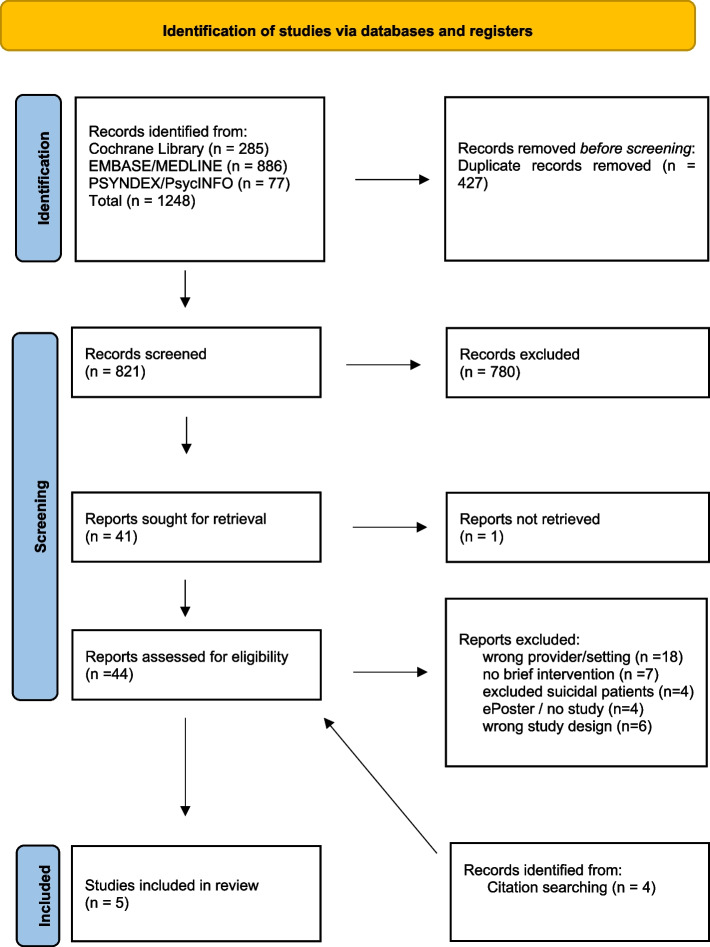
PRISMA Flow Diagram adapted from Page et al. (2021) [33]

In total, this systematic review identified *N* = 1252 relevant studies. Of these, *N* = 1248 were sourced from the electronic database search, and *N* = 4 were identified through manual literature researches. After duplicate removal, title and abstract screening was conducted on the remaining 825 studies, resulting in the exclusion of 781 records. Subsequently, 44 studies underwent full-text assessment, with 39 studies ultimately excluded for specific reasons. The primary reasons for exclusion were misclassification of “provider” and lacking briefness regarding the intervention. Figure 1 provides a detailed overview of the study selection process. Ultimately, five studies met the inclusion criteria and were incorporated into this systematic review.

### Risk of bias

**Fig. 2:**
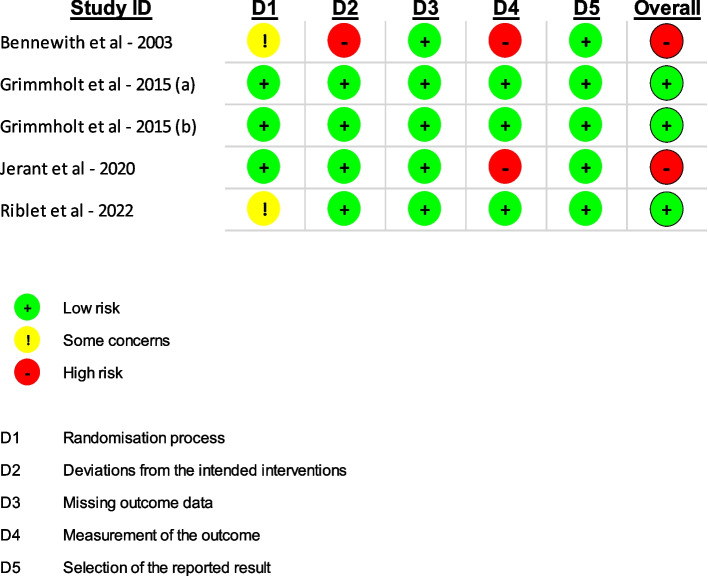
ROB-2 results

Figure 2 illustrates the risk of bias assessment using the ROB-2 tool.

The study by Bennewith et al. (2002) [42] shows a high risk of bias due to deviations from the intended intervention, issues in outcome measurement, and potential sources of bias in the randomization process, resulting in an overall high risk of bias. In contrast, the studies by Grimholt et al. (2015) [43, 44] demonstrated only a low risk of bias. Jerant et al. (2020) [45] exhibited a high risk of bias, particularly in outcome measurement, contributing to an overall elevated bias risk. While Riblet et al. (2022) [46] identified some concerns in the randomization process, the overall risk of bias remained low.

### Quality of evidence

The GRADE methodology [40, 41] indicated low certainty of evidence, primarily due to significant heterogeneity in the outcomes of the included studies, which resulted from varying interventions and measured outcomes. Concerns regarding the directness and precision of the evidence were also prominent. In particular, substantial differences in study populations significantly limit the generalizability of the findings [47]. Additionally, the small sample sizes across the studies likely affected the robustness and reliability of the results.

### Study characteristics

Table 1 provides an overview of the studies included in this systematic review. In addition, Table 2 provides an overview of included interventions.

A total of 2202 participants were included in the review, with demographic data available for 2149 participants. Of those, 895 were male (41.65%) and 1254 were female (58.35%). The age of participants ranged from 16 to 95 years, with a mean age of 44 years.

**Table 1:** Overview of studies included in this systematic review

**Author**	**Year, Country**	**Total Intervention Duration**	**Duration of Individual Patient Contacts**	**Provider**	**Randomised Participants**	**GP Practices**	**IG**	**CG**	**Mean age in years (range)**	**Men (***n***,%)**	**Women (*n*,%)**
Bennewith et al. [42]	2002, UK	12 months	-	GP	1932	98	964	968	32.55 (16 to 95)	796 (41.2%)	1136 (58.8%)
Grimholt et al. [43, 44]	2015, Norway	6 months	-	GP	202	-	62	87	37.8 (18 to 75)	38 (25.5%)	111 (74.5%)
Jerant et al. [45]	2020, US	15–20 min	One contact	GP	48	42	21	27	55.9 (35–74)	48 (100%)	0 (0%)
Riblet et al. [46]	2022, US	3 months	Initial contact: 60 min, six follow-ups up to 30 min	Psychologist, Social Worker, or Nurse	20	-	10	10	51.5 (≥ 18)	13 (65%)	7 (35%)

*IG* Intervention group, *CG* Control group, *GP* General Practitioner, *UK* United Kingdom, *US* United States

**Table 2:** Detailed description of interventions

Author	Description of the Intervention
Bennewith et al. [42]	The intervention aimed to reduce the recurrence of self-harm episodes. Each week, patients presenting with new self-harm incidents within intervention-arm practices were identified via a UK self-harm registry. For first-time incidents, the general practitioner (GP) was notified and provided with a letter to invite the patient for consultation. The GP also received evidence-based guidelines, developed through a modified Delphi method, to standardize the management of self-harm. This approach ensured timely and structured care, enabling individualized counseling and initiation of appropriate therapeutic interventions
Grimholt et al. [43, 44]	The intervention aimed to reduce suicidal ideation and improve follow-up attendance after intentional self-poisoning. Conducted by the general practitioner (GP) over six months, it included an initial consultation one-week post-discharge, followed by monthly appointments for three months and two additional sessions six weeks apart. The structured consultations, based on WHO guidelines and expert consensus, focused on three key areas: 1. Timely Scheduling: Arrange a follow-up within one-week post-discharge 2. Patient Assessment: Address reasons for self-poisoning, primary concerns, suicidal ideation, current treatment, and support needs 3. Follow-up Planning: Schedule additional sessions to ensure consistent care and support
Jerant et al. [45]	The intervention aimed to activate middle-aged men with suicidal ideation to discuss these thoughts with their general practitioners. This was facilitated through the MAPS program (Men and Providers Preventing Suicide), an interactive, 15–20-min computer-based tool designed to encourage disclosure during clinic visits. The program included multimedia modules addressing common barriers and provided guidance on (1) discussing suicidal thoughts with a GP, (2) developing a personalized treatment plan, and (3) monitoring and adjusting the plan in collaboration with the care team
Riblet et al. [46]	The study aimed to evaluate the reduction of suicidal ideation and the feasibility of implementing the “Veterans Affairs Brief Intervention and Contact Program” (VA BIC) within an integrated care system. Modeled on the “World Health Organization Brief Intervention and Contact Program” (WHO BIC) [48], originally developed to prevent post-discharge suicide attempts, the VA BIC adapted to pandemic conditions through video and telephone contacts. The intervention commenced with a one-hour session covering suicide prevention strategies, safety planning, and social support, followed by up to six 30-min follow-up contacts

A total of 2202 participants were included in the review, with demographic data available for 2149 participants. Of those, 895 were male (41.65%) and 1254 were female (58.35%). The age of participants ranged from 16 to 95 years, with a mean age of 44 years.

### Main outcomes

Table 3 provides an overview of the main outcomes and results of the studies included in this systematic review.

**Table 3:** Outcome and results of included studies

Author	Outcome	IG (95% CI)	CG (95% Cl)	*p*-Value
Bennewith et al. [42]	Primary Outcome: Incidence of repeated episodes of intentional self-harm	Incidence rate ratio 1,24 (0,92–1,68)		*p* = 0,16
	Secondary Outcome: Time to repeated episode of intentional self-harm	HR: 1,15 (0,94–1,42)		*p* = 0,17
	Subgroup Analysis: Patients with a history of self-harm	OR: 0,57 (0,33 – 0,98)		*p* = 0,0017
	Subgroup Analysis: Patients without a history of self-harm	OR: 1.32 (1.02 to 1.70)		*p* = 0,0017
	Process Data: Patients with a GP consultation within 6 weeks	58% (IG) vs. 56.9% (CG)		
Grimholt et al. [44] Grimholt et al. [43]	Primary Outcome: SSI	(13.3–18.8)	(14.6–19.9)	*p* = 0,52
	Secondary Outcome: BDI	(23.1–29.0)	(21.7–27.0)	*p* = 0,4
	Secondary Outcome: BHS	(8.7–11.4)	(8.7–11.2)	*p* = 0,94
	Secondary Outcome: BSI	(11.3–13-,9)	(10,9–14,1)	*P* = 0,92
	Outcome	IG (%)	CG (%)	
	Satisfaction with GP’s attentiveness to personal issues	93,1	59,4	*p* = 0,002
	Satisfaction with SDM	87,5	54,8	*p* = 0,009
	Satisfaction with treatment (at 3 months)	83	68	*p* = 0,158
	Satisfaction with treatment (at 6 months)	79	51	*p* = 0,026
		IG (mean)	CG (mean)	
	Contacts with the general practitioner during the study period	6,7	4,5	*p* = 0,005
Jerant et al. [45]	Primary Outcome: Discussion of suicidal thoughts during general practitioner appointment	OR: 5.91 (1,59–21,94)		*p* = 0,008
	Effect among men with preparatory suicidal thoughts	OR: 27,45 (2,74–274,96		*p* = 0,005
	Effect among men without preparatory suicidal thoughts	OR: 2,10 (0,27–16,59		*p* = 0,48
Author	Calculated effect sizes for the VA-BIC intervention combined with standard care compared to standard care alone	Cohen’s d	Hedges’ g	Benefit for IG
Riblet et al. [46]	SSI			
	after 1 month	-0,38	-0,37	Yes
	after 3 months	-0,74	-0,71	Yes
	BHS			
	after 1 month	-0,18	-0,17	Yes
	after 3 months	-0,62	-0,60	Yes

*BDI* Beck Depression Inventory, *BHSBeck* Hopelessness Scale, *BSI* Beck Suicidal Intent Scale, *CG* Control group, *CI* Confidence interval, *GP* General Practitioner, *HR* Hazard ratio, *IG* Intervention group, *OR* Odds ratio, *SDM* Shared decision making, *SSI* Beck Scale for Suicide Ideation

The intervention by Bennewith et al. (2002) [42], aimed at reducing repeated self-harm episodes through GP-based care, did not achieve a statistically significant reduction in recurrence. The primary outcome indicated a slightly higher repetition rate in the intervention group versus the control group.

Grimholt et al. (2015) [43, 44] assessed outcomes via the Beck Scale for Suicide Ideation (BSS) [49], Beck Depression Inventory (BDI) [50], the Beck Hopelessness Scale (BHS) [51], and patient satisfaction measured using the EUROPEP scale. No significant differences were observed between groups on these scales at three or six months. However, self-reported reoccurrence of self-poisoning was significantly higher in the intervention group (39.5%) compared to the control group (15.8%, *p* = 0.009).

Jerant et al. (2020) [45] demonstrated that men participating in the Men and Providers Preventing Suicide (MAPS) program were significantly more likely to discuss suicidal thoughts with their GP. Logistic regression indicated that MAPS participants were nearly six times more likely to engage in these discussions than controls, with an even stronger effect among those with preparatory suicidal behaviours.

Riblet et al. (2022) [46] evaluated the “Veterans Affairs Brief Intervention and Contact Program” (VA BIC) within a virtual integrated care system. Among the 20 enrolled participants, 95% (*n* = 19) completed all assessments, with 90% (*n* = 9) of the intervention group attending all scheduled sessions, and all meeting at least 70% attendance. Findings indicated a positive trend in reducing suicidal ideation and hopelessness, especially at three months, with moderate-to-large effect sizes. Improvements in treatment adherence and social connectedness were observed in the intervention group, though these effects were less pronounced. Notably, patients in the control group demonstrated greater improvements in specific domains, including coping ability and social connectedness.

## Discussion

This systematic review identified five publications investigating brief interventions for suicidality in primary care, encompassing four distinct interventions [42–46]. The findings highlight a critical gap in the literature, as most brief interventions for suicidality are designed for emergency settings rather than primary care [9, 52–57]. Given that general practitioners (GPs) are often the first point of contact for patients with mental health concerns, including suicidality, this setting is crucial. Long-term relationships with GPs allow for early detection of subtle changes in patients’ behaviour or mental state that may indicate suicide risk, especially in areas lacking specialized mental health services [58–61].

Methodologically, several interventions in this review exhibit strengths in design, particularly in structuring support for patients experiencing suicidality. For instance, Bennewith et al. (2002) employed a Delphi-method-based approach to develop evidence-based guidelines for GPs [62]. The Delphi method has a wide range of potential uses in mental health research [63], specifically in suicide research [64, 65]. Similarly, in the study by Grimholt et al. (2015), GPs received short guidelines developed from the WHO guide to general practice [66]. Despite these structured approaches, neither study demonstrated a significant benefit for patients in the intervention group. Jerant et al. (2020) specifically targeted men and used motivational elements to address barriers in discussing suicidal thoughts, an approach supported by prior health communication and suicide prevention research [67–69]. Men participating in the intervention programme were significantly more likely to discuss suicidal thoughts with their GP, indicating a need for tailored approaches [70, 71].

Furthermore, intervention duration and follow-up structure appear to play a crucial role in outcomes. Time is a valuable resource in primary care, requiring interventions to be both time- and staff-efficient. While studies in clinical settings have demonstrated the effectiveness of brief interventions with follow-up contacts for high-risk populations [48], robust evidence for their efficacy in primary care remains lacking. This highlights the need for innovative models, such as the integrated telehealth-based approach seen in Riblet et al. (2022), which may offer a feasible alternative to enhance patient follow-up while minimising resource burden on primary care providers.

Our review identified a lack of effective brief interventions in primary care for patients with suicidal ideation, highlighting an urgent need for research. Based on our systematic literature review, we recommend the following key elements for inclusion in future interventions to enhance the role of primary care in suicide prevention:

- Motivational Components, including Motivational Interviewing: Motivational Interviewing (MI) is a well-established therapeutic approach shown to be effective in encouraging behavioural change across a variety of mental health conditions [72]. As highlighted in Riblet et al. (2022) and other studies, MI can be particularly promising for suicide prevention [73–76] and is suitable for use in general practice [74, 77]. Future research should focus on refining the application of MI for suicide prevention in general practice, considering its adaptation for different at-risk populations such as adolescents, older adults, or individuals with co-occurring mental health disorders, and explore MI integration with other therapeutic methods, such as Cognitive Behavioural Therapy (CBT) [78].
- Safety Planning: Safety planning interventions are a cornerstone of suicide prevention [79]. A critical component in Riblet et al. (2022), safety planning should be part of future primary care interventions [9, 80] ensuring its accessibility for patients with varying levels of health literacy and socio-economic backgrounds. Studies could investigate the role of digital tools, such as apps or online platforms, in facilitating the creation and updating of safety plans [81–83]. However, apps should be seen as an addition to an ongoing patient–provider relationship, never as a replacement.
- Structured, Regular Patient Contacts: Structured and regular follow-ups are a critical element of effective suicide prevention, especially when delivered within frameworks such as the Chronic Care Model (CCM) and the Collaborative Care Model (COCM) [84–86]. Future research could investigate the impact of different models of care delivery, such as remote monitoring by non-GP staff or virtual consultations, on patient outcomes. The inclusion of non-GP staff in follow-up care could allow for more frequent contact with patients, providing consistent support and fostering a sense of continuity in care [87]. Research on the use of virtual or remote care models [82], particularly in rural or underserved areas, could offer insights into how such interventions can be scaled and tailored to diverse patient populations. Our findings are in line with previous research emphasising the importance of safety planning and the continuity of care [64, 65]. A brief intervention complements, rather than replaces, treatment, helping healthcare providers encourage behaviour change. Primary care offers unique opportunities to identify and address modifiable risk factors, with multidisciplinary teams providing personalised advice, follow-up, and referrals as needed [88].

### Strengths and limitations

The greatest strength of this review is its focus on brief interventions for suicide prevention in primary care. A systematic approach was used to identify relevant studies, with all search terms, operators, databases, and filters documented for transparency and replicability. The ROB 2 tool provided a standardised method to evaluate bias in RCTs, ensuring comprehensive and reproducible quality assessments.

As a limitation, there is no universally accepted definition of a “brief” intervention. While one study may define brief interventions as a single, short counselling session, another might include multiple sessions over several weeks. This variability complicates the comparison and synthesis of results. Furthermore, this inconsistency introduces the risk of systematic bias. Studies with differing definitions may be disproportionately included or excluded during selection, which could compromise the validity of the review and lead to erroneous conclusions. The included interventions varied in design, target population, and effectiveness. Finally, the reviewed studies exhibit varying levels of bias, primarily due to issues with randomization, blinding, and intervention fidelity. The overall evidence quality is very low due to inconsistencies, heterogeneous interventions, and small sample sizes, limiting generalizability.

## Conclusion

This review underscores the need for more research to adapt brief interventions to enhance the role of primary care in suicide prevention. Combining motivational elements, safety planning, structured follow-ups, and collaborative care models appears promising for developing effective suicide prevention strategies. Ultimately, while brief interventions hold promise, more research effort is required to refine, validate, and integrate these strategies into everyday clinical practice.

## Supplementary Information

Supplementary Material 1.

Supplementary Material 2.

## Data Availability

The data generated in the current study are available from the corresponding author on reasonable request. However, restrictions may apply to the availability of the underlying original articles: If they are not open access, they may be subject to restrictions.
