# Supplementary material for: Brief interventions for suicidal ideation in primary care: a systematic review
Source: BMC Prim Care. 2025 May 15;26:167. doi: 10.1186/s12875-025-02848-4 (PMC12080141; doi:10.1186/s12875-025-02848-4)
Supplement: Supplementary file 1 — Supplementary Material 1. [file 12875_2025_2848_MOESM1_ESM.docx]

Supplement: Search Strategy

## Cochrain Suchstrategie via Cochrain Library

| # | Searches | Results |
| --- | --- | --- |
| 1 | ("primary care doctor" OR "primary care doctors" or "primary care physician" OR "primary care physicians" or "primary medical care" OR "primary care” OR "primary health care” OR "primary healthcare" OR "general practitioner" OR "general physician" OR "general practice physician" OR "general practice physicians" or "general medical practice" OR "general medical practices" or "general medicine" OR "family doctor" OR "family doctors" or "family physician" OR "family physicians" or "family practitioner" or "family practitioners" "family practice" OR "family practitioner“ OR "gp“) | 45614 |
| 2 | MeSH descriptor: [Primary Health Care] explode all trees | 10429 |
| 3 | MeSH descriptor: [Physicians, Primary Care] explode all trees | 214 |
| 4 | MeSH descriptor: [Physicians, Family] explode all trees | 511 |
| 5 | MeSH descriptor: [General Practitioners] explode all trees | 494 |
| 6 | MeSH descriptor: [General Practice] explode all trees | 3046 |
| 7 | MeSH descriptor: [Family Practice] explode all trees | 2246 |
| 8 | MeSH descriptor: [Ambulatory Care] explode all trees | 4139 |
| 9 | #1 or #2 or #3 or #4 or #5 or #6 or #7 or #8 | 4139 |
| 10 | ("suicide" or “suicides” or "self inflicted death" or "self-inflicted death" or "self-harm" or “self harm" or “self injury" or "self injuries" or ”self destructive behaviour" or ”self-destructive behaviour" or “behaviors self-destructive” or “behaviors self-injurious” or “deliberate self harm” or "suicide prevention" or "Self-Injurious Behavior") | 43861 |
| 11 | MeSH descriptor: [Suicide] explode all trees | 6171 |
| 12 | MeSH descriptor: [Suicidal Ideation] explode all trees | 2192 |
| 13 | MeSH descriptor: [Suicide Prevention] explode all trees | 1048 |
| 14 | MeSH descriptor: [Self-Injurious Behavior] explode all trees | 441 |
| 15 | #10 or #11 or #12 or #13 or #14 | 2485 |
| 16 | MeSH descriptor: [Clinical Trial] explode all trees | 6468 |
| 17 | MeSH descriptor: [Controlled Clinical Trial] explode all trees | 45347 |
| 18 | MeSH descriptor: [Randomized Controlled Trial] explode all trees | 38477 |
| 19 | (“clinical trial” or “randomized controlled trial” or “Controlled Clinical Trial” or “RCT”) | 25732 |
| 20 | 17 or 18 or 19 or 20 | 787636 |
| 21 | #9 and #15 and #20 (limit: Jan 2000 – Oct 2023) | 285 |
| 12 |  |  |

## Medline und EMBASE Suchstrategie via OVID

| # | Searches | Results |
| --- | --- | --- |
| 1 | ("primary care doctor*" OR "primary care physician*" OR "primary medical care" OR "primary care” OR "primary health care” OR "primary healthcare" OR "general pract*" OR "general physician*" OR "general practice physician*" OR "general medical practice*" OR "general medicine" OR "family doctor*" OR "family physician*" OR "family pract*" OR "family practitioner“ OR "gp“).mp. | 879486 |
| 2 | exp general practitioner/ or exp primary medical care/ or exp primary health care/ | 495521 |
| 3 | 1 OR 2 | 973941 |
| 4 | ("suicid*" or "self inflicted death*" or "self-harm*" or “self harm" or “self injur*" or ”self destructive behaviour" or “self-destructive" or “behaviors self-destructive” or “behaviors self-injurious” or “deliberate self harm”).mp. | 287180 |
| 5 | exp suicide/ or exp suicide attempt/ or exp suicidal ideation/ exp Self-Injurious Behavior/ | 207284 |
| 6 | 4 or 5 | 295696 |
| 7 | exp randomized controlled trial/ | 1373814 |
| 8 | (clinical trial or randomized controlled trial or Controlled Clinical Trial).mp. | 3355609 |
| 9 | 7 OR 8 | 3356750 |
| 10 | 3 AND 6 AND 9 | 934 |
| 11 | limit 10 to yr="2000 -Current" | 897 |
| 12 | limit 11 to english language | 886 |

## PSYNDEX und PsycINFO Suchstrategie via EBSCO

| # | Searches | Results |
| --- | --- | --- |
| 1 | TI ("primary care doctor*" OR "primary care physician*" OR "primary medical care" OR "primary care” OR "primary health care” OR "primary healthcare" OR "general pract*" OR "general physician*" OR "general practice physician*" OR "general medical practice*" OR "general medicine" OR "family doctor*" OR "family physician*" OR "family pract*" OR "family practitioner“ OR "gp“) | 16,983 |
| 2 | AB ("primary care doctor*" OR "primary care physician*" OR "primary medical care" OR "primary care” OR "primary health care” OR "primary healthcare" OR "general pract*" OR "general physician*" OR "general practice physician*" OR "general medical practice*" OR "general medicine" OR "family doctor*" OR "family physician*" OR "family pract*" OR "family practitioner“ OR "gp“) | 46,140 |
| 3 | MM ("Primary Health Care" OR "General Practitioners" OR "Family Physicians" OR "General Practitioners" OR "Internists" OR "Family Medicine" OR OR "Counseling" OR "Suicide Prevention Centers" ) | 19,243 |
| 4 | s1 or s2 or s3 | 50,188 |
| 5 | TI ("suicid*" or "self inflicted death*" or "self-harm*" or “self harm" or “self injur*" or ”self destructive behaviour" or “self-destructive" or “behaviors self-destructive” or “behaviors self-injurious” or “deliberate self harm” OR "Military Suicide") | 38,077 |
| 6 | AB ("suicid*" or "self inflicted death*" or "self-harm*" or “self harm" or “self injur*" or ”self destructive behaviour" or “self-destructive" or “behaviors self-destructive” or “behaviors self-injurious” or “deliberate self harm”) | 64,605 |
| 7 | MM ("Suicidal Behavior" OR "Attempted Suicide" OR "Suicidal Ideation" OR "Suicide" OR "Suicidal Ideation" OR "Suicidality" OR "Suicide" OR "Suicide Prevention" OR "Self-Inflicted Wounds" OR "Self-Mutilation" OR "Self-Poisoning" OR "Self-Injurious Behavior" OR "Self-Inflicted Wounds" OR "Attempted Suicide" OR "Self-Destructive Behavior" OR "Nonsuicidal Self-Injury" OR "Self-Poisoning") | 40,554 |
| 8 | s5 or s6 or s7 | 66,650 |
| 9 | TI ("RCT“ OR " randomized controlled trial*" OR "Controlled Trial*" OR “double blinded trail*”) | 20,648 |
| 10 | AB ("RCT“ OR " randomized controlled trial*" OR "Controlled Trial*" OR “double blinded trail*”) | 43,468 |
| 11 | MM ("Randomized Controlled Trials" OR "Randomized Clinical Trials" OR "Clinical Trials") | 7,460 |
| 12 | s9 or s10 or s11 | 57,325 |
| 13 | s4 and s8 and s12 (from 2000-2023 | 77 |
